# Linear Relationship between Hepatic Steatosis Index and Major Adverse Cardiovascular Events in Hypertensive Patients with Obstructive Sleep Apnea: A Real-World Cohort Study from China

**DOI:** 10.31083/j.rcm2410280

**Published:** 2023-10-07

**Authors:** Wen Wen, Xintian Cai, Qing Zhu, Junli Hu, Jing Hong, Xiangyang Zhang, Nanfang Li

**Affiliations:** ^1^Hypertension Center of People’s Hospital of Xinjiang Uygur Autonomous Region, Xinjiang Hypertension Institute, NHC Key Laboratory of Hypertension Clinical Research, Key Laboratory of Xinjiang Uygur Autonomous Region “Hypertension Research Laboratory”, Xinjiang Clinical Medical Research Center for Hypertension (Cardio-Cerebrovascular) Diseases, 830000 Urumqi, Xinjiang Uygur Autonomous Region, China; ^2^The First Affiliated Hospital of Xinjiang Medical University, 830000 Urumqi, Xinjiang Uygur Autonomous Region, China

**Keywords:** hypertension, obstructive sleep apnea, hepatic steatosis index, cardiovascular disease, cohort

## Abstract

**Background:**

Hypertensive patients with obstructive sleep apnea (OSA) are at a high risk of cardiovascular disease (CVD), but assessments of CVD risk in this population are frequently constrained by the presence of comorbid medical conditions. The noninvasive and convenient hepatic steatosis index (HSI) can not only predict the degree of fatty liver degeneration but also correlates well with the severity of numerous diseases. However, the relationship between the HSI and CVD in hypertensive patients with OSA remains unclear.

**Methods:**

This retrospective cohort study included patients aged ≥18 years with hypertension and a primary diagnosis of OSA and grouped them according to their baseline HSI. The primary outcome was new or recurrent major adverse cardiovascular and cerebrovascular events (MACCE), while the secondary outcomes were cardiac and cerebrovascular events. The relationship between the baseline HSI and the risk of endpoint events was evaluated using Kaplan–Meier curves, risk-factor graphs, and Cox regression models, while generalized additive models were used to identify linear relationships. The C-statistic, integrated discrimination improvement (IDI), and net reclassification index (NRI) were used to evaluate the predictive value of HSI increments for endpoint events.

**Results:**

A total of 2467 participants were included in the analysis and separated into four groups (Q1–Q4) based on their HSI quartiles. Kaplan–Meier survival curves indicated that patients in the Q4 group had the lowest survival time. The Q4 group also showed a significantly higher risk of MACCE (HR [hazard ratio], 2.95; 95% CI [confidence interva]: 1.99–4.39; *p <* 0.001), cardiac events (HR, 2.80; 95% CI: 1.68–4.66; *p <* 0.001), and cerebrovascular events (HR, 3.21; 95% CI: 1.71–6.03; *p <* 0.001). The dose-response curve revealed a linear association between the HSI and the occurrence of endpoint events. For every unit increase in the HSI, the risks of MACCE, cardiac events, and cerebrovascular events increased by 43%, 38%, and 51%, respectively. The C-statistic, IDI, and NRI all indicated that the model including the HSI showed better discriminatory and classification efficacy for endpoint events in comparison with the conventional model (*p <* 0.05).

**Conclusions:**

The HSI showed a linear relationship with the risk of MACCE in hypertensive OSA patients.

## 1. Introduction 

The number of people with hypertension worldwide in 2021 was estimated to be 1.4 billion [1], and the prevalence of obstructive sleep apnea (OSA) among hypertensive patients is 40% [2]. Hypertensive patients with OSA usually show a high risk of cardiovascular disease (CVD). In comparison with patients showing hypertension or OSA alone, those with both conditions are more susceptible to CVD and frequently experience more severe adverse cardiovascular events [3]. In addition, because OSA often occurs during nighttime sleep and is not easily noticed by patients, its prevalence is heavily underestimated [4]. Therefore, in addition to paying more attention to the population with hypertension and OSA, assessments of CVD prognosis in this population also need to be strengthened.

The pathophysiological mechanisms underlying OSA-induced increments in blood pressure can be summarized as follows: First, intermittent hypoxia caused by OSA leads to systemic oxidative stress, resulting in increased endothelin-1 production and decreased nitric oxide production in endothelial cells and causing increased peripheral arterial resistance [5, 6]. Second, recurrent episodes of hypoxemia and fragmented sleep induce sympathetic excitation, resulting in increased cardiac output and peripheral vasoconstriction [7]. Third, intermittent hypoxia can increase renin production through renal sympathetic excitation and thereby increase the levels of plasma angiotensin-II, which shows vasoconstrictor activity, and aldosterone, which shows sodium- and water-retention effects [8]. Fourth, intermittent hypoxia can result in vascular endothelial dysfunction and aberrant lipid metabolism, both of which are linked to atherosclerosis [9, 10]. All of these factors can contribute to hypertension, and the increased blood pressure can exacerbate these pathophysiological processes [9, 10, 11]. The pathophysiological mechanisms underlying OSA and hypertension overlap and reinforce one another, and the resulting cardiovascular damage is significantly greater than that caused by hypertension or OSA alone. Therefore, patients with both conditions constitute a high-risk CVD group that requires attention.

The assessment of groups showing high cardiovascular risk is currently based on the domestic and international clinical recommendations for CVD. However, in addition to necessitating comprehensive evaluations by professional physicians with extensive clinical experience, this approach also requires a number of tests and assessments of multiple inspection indicators as the basis for evaluation, limiting these evaluations to patients hospitalized in high-level hospitals. In China, however, a majority of the substantial population of patients with hypertension and OSA reside in rural areas with poor medical conditions, so they cannot be tested professionally. In addition, the evaluation group targeted by the clinical recommendations for CVD in China and overseas does not include patients with hypertension and OSA, and evaluations of this population lack specificity. These aspects indicate the urgent need for a practical, inexpensive, noninvasive, measurable, and more relevant methods for predicting and assessing the risk of CVD in hypertensive individuals with OSA.

The hepatic steatosis index (HSI), which is calculated using the formula HSI = 8 (alanine aminotransferase [ALT]/aspartate aminotransferase [AST] ratio) + body mass index (BMI) (+2, if female; +2, if diabetes) was originally used to evaluate hepatic steatosis [12]. While the HSI can effectively evaluate hepatic steatosis (its sensitivity and specificity for diagnosing nonalcoholic fatty liver disease are both above 90%), it has been also shown to be related to the severity of diseases such as type 2 diabetes, CVD, and organ injuries unrelated to hypertension [13, 14, 15, 16]. The aforementioned findings indicate an association between the HSI and other diseases, and suggest that this association is related to a high metabolic risk in the population. Although patients with OSA and hypertension are more likely to develop metabolic disorders [17, 18, 19, 20, 21], to our knowledge, no previous study has evaluated the relevance of the HSI in hypertensive patients with OSA. Thus, the purpose of this study was to use the HSI to assess the risk of major adverse cardiovascular and cerebrovascular events (MACCE) in hypertensive individuals with OSA.

## 2. Materials and Methods

### 2.1 Research Population

**Fig. 1.**
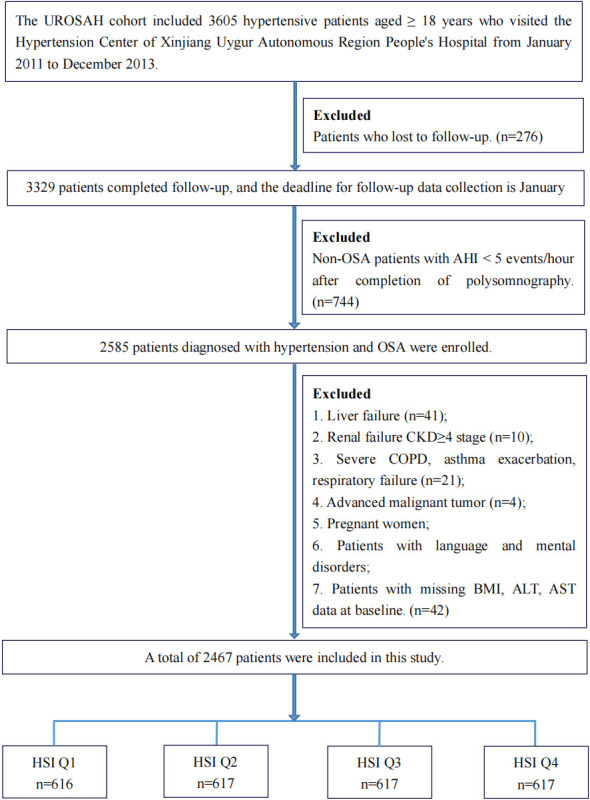
**Flowchart of the study participants.** Abbreviations: HSI, hepatic steatosis index; UROSAH, Urumqi Research on Sleep Apnea and Hypertension cohort; OSA, obstructive sleep apnea; AHI, apnea–hypopnea index; CKD, chronic kidney disease; COPD, chronic obstructive pulmonary disease; BMI, body mass index; AST, aspartate transaminase; ALT, alanine transaminase.

The data for this investigation were obtained from the Urumqi Research on Sleep Apnea and Hypertension (UROSAH) study, a retrospective cohort study of 3605 hypertensive patients at the Hypertension Center of People’s Hospital of Xinjiang Uygur Autonomous Region. The inclusion and exclusion criteria and design details of this cohort have been reported previously [22, 23]. A total of 3329 participants aged >18 years agreed to participate in this study and completed the follow-up (loss rate, 7.65%). We excluded 744 patients without OSA whose apnea–hypopnea index (AHI) was <5 after polysomnography (PSG); 41 patients with severe hepatic insufficiency, liver malignancy, cirrhosis, chronic viral hepatitis, or elevated ALT and AST levels caused by other extrahepatic causes; 25 patients with chronic kidney disease (CKD) ≥ stage 4, severe chronic obstructive pulmonary disease, acute attack of asthma, respiratory failure, advanced cancer, language disorders, or active pregnancies; and 42 participants with missing values for BMI and ALT and AST levels in baseline data. Thus, a total of 2467 participants were included in the study analysis (Fig. 1). This study was conducted after obtaining the consent of all participants and was approved by the Medical Ethics Committee of the People’s Hospital of Xinjiang Uygur Autonomous Region (ethical approval number: 2019030662). The study strictly adhered to the ethical standards of the Declaration of Helsinki.

### 2.2 Data Collection and Definition

The baseline data collected included demographic information, previous medical history, medication history, and laboratory test findings. The baseline anthropometric measurements and laboratory testing procedures have been described previously [22]. OSA was defined by a sleep AHI ≥5 times/hour, while OSA severity was defined as mild (5 ≤ AHI < 15), moderate (15 ≤ AHI < 30), or severe (AHI ≥30) [24]. Hypertension was defined by a resting blood pressure systolic blood pressure ≥140 mmHg and/or diastolic blood pressure ≥90 mmHg or current use of antihypertensive drugs [25]. Diabetes was diagnosed on the basis of the diagnostic criteria of the 2013 edition of the Chinese Guidelines for the Prevention and Treatment of Diabetes [26]. HSI was determined using the formula 8 (ALT/AST ratio) + BMI (+2 if females; +2 if diabetes). Smoking and alcohol consumption habits were categorized as never, past, or present.

### 2.3 Follow-Up and Outcomes

Follow-up assessments were conducted by telephone, and the outpatient and inpatient medical records of all patients were collected. Patients were followed up until the occurrence of any of the endpoints of the study or until the end of the follow-up period in January 2021, whichever occurred first. The collected data included the latest blood pressure level, all clinical examination results, and the appearance of new or recurrent MACCE. MACCE were evaluated by collecting the inpatient medical records of the participants and were judged by more than two blinded high-level clinical experts. For deaths, the cause of death was determined from relatives of the deceased and verified by hospital death certificates, hospitalization data, or the local public security system. Illnesses (including death) and surgical procedures were classified using the 10th revision International Classification of Diseases codes (ICD-10) and clinical modification codes for procedures and procedures of the 9th revision of the International Classification of Diseases (ICD-9-CM-3). In this study, MACCE included nonfatal myocardial infarction (ICD-10 codes: I21.0–I21.4, I21.9, I22), coronary and cerebrovascular revascularization (ICD-9-CM-3 codes: 36.03, 36.04, 36.06, 36.07, 36.09, 36.11-36.14, 36.2, 36.3, 38.11, 38.12, 38.31, 38.32, 38.41, 38.42, 39.74, and 39.76), unstable angina (I20) or rehospitalization for heart failure (I11.0, I13.0, I13.2, I50.0, I50.1, I50.9), cardiac death, nonfatal stroke (I61, I62.0, I62.9, I63, I64, excluding I63.801), and brain-derived death. Cardiac events were defined as nonfatal myocardial infarction, revascularization, rehospitalization for unstable angina or heart failure, or cardiac death. Cerebrovascular events were defined as nonfatal stroke or brain-derived death. All endpoints were defined in accordance with the suggested definitions for standardized data-collection schemes in cardiovascular trials [27].

### 2.4 Treatment Management

At discharge, patients were provided an individualized treatment plan and instructed to take their medications on time, consume a healthy diet, control their weight, increase their physical activity level appropriately, and self-measure their blood pressure daily at home. Patients with moderate-to-severe OSA at baseline were advised to use a continuous positive airway pressure (CPAP) ventilator, and those who consented to CPAP therapy received an individualized formulation for CPAP therapy. During outpatient, inpatient, or telephone follow-up visits, we evaluated the patients’ current conditions and recommended lifestyle, medication, blood pressure monitoring, and weight modifications. However, we did not obtain follow-up data on the CPAP therapy administered to the patients.

### 2.5 Statistical Analysis

Continuous variables were reported using mean and standard deviation (SD) or median and interquartile range (IQR), and analysis of variance and Kruskal–Wallis H tests were used to compare groups. Categorical variables were expressed as numbers and percentages, and chi-square tests were used for comparisons between groups. Kaplan–Meier survival curves were used to estimate survival probability for the primary outcome, and the log-rank test was performed to analyze differences in survival between HSI groups. Potential connections between the HSI and endpoint events were identified using generalized additive models, while independent predictors of endpoint events were identified using univariate and multivariate Cox proportional-hazard models. On the basis of the initial model, we created two more models to account for various confounding factors. Model 1 was adjusted for age and sex. Model 2 was further adjusted for systolic blood pressure, diastolic blood pressure, heart rate, smoking and alcohol consumption status, homocysteine (Hcy) level, OSA grade, and diabetes history (with or without). The C-statistic, integrated discrimination improvement (IDI), and net reclassification index (NRI) were obtained to assess the prediction ability of HSI increments for endpoint events. Data were analyzed using R (version 4.1.1, R Foundation for Statistical Computing, Vienna, Austria) statistical software; all analyses were two-tailed, and *p <* 0.05 was considered statistically significant.

## 3. Results

### 3.1 Participant Characteristics

Participants were divided into four groups (Q1–Q4) according to HSI quartiles, and the average HSI value in groups Q1, Q2, Q3, and Q4 was 32.74 ± 2.29, 37.43 ± 1.09, 41.22 ± 1.18, and 47.53 ± 3.58, respectively. Table 1 shows the baseline characteristics of all participants from Q1–Q4. Participant age showed a reducing trend from Q1 to Q4 (53.35 ± 11.58, 50.78 ± 9.94, 48.25 ± 9.82, and 46.15 ± 10.48 years). BMI, neck circumference, waist circumference, creatinine level, the proportions of smokers and patients consuming alcohol, and the proportion of patients with a history of diabetes increased gradually across the groups, with the differences showing statistical significance (*p <* 0.05). The distribution of patients with a history of cerebrovascular disease at baseline showed a decreasing trend among the four groups, and the proportion of patients with a history of CVD at baseline, the duration of hypertension, AHI, OSA severity, and medication history did not show statistically significant differences among the four groups. During the mean follow-up period of 6.45 years, the total number of MACCE was 53 (8.60%), 77 (12.48%), 86 (13.94%), and 140 (22.69%) in groups Q1, Q2, Q3, and Q4, respectively; the number of cardiac events was 31 (5.03%), 47 (7.62%), 54 (8.75%), and 91 (14.75%), respectively; and the number of cerebrovascular events was 22 (3.57%), 30 (4.86%), 32 (5.19%), and 49 (7.94%), respectively.

**Table 1.** **Baseline characteristics of the participants categorized by HSI quartiles**.

Variable		Quartile 1	Quartile 2	Quartile 3	Quartile 4	*p*-value
		(23.42–35.49)	(35.5–39.27)	(39.28–43.42)	(43.43–68.25)	
		n = 616	n = 617	n = 617	n = 617	
Age, years		53.35 ± 11.58	50.78 ± 9.94	48.25 ± 9.82	46.15 ± 10.48	<0.001
Sex n (%)						0.019
	Female	221 (35.88%)	200 (32.41%)	184 (29.82%)	173 (28.04%)	
	Male	395 (64.12%)	417 (67.59%)	433 (70.18%)	444 (71.96%)	
BMI, ${\mathrm{kg/m}}^{2}$		24.73 ± 2.15	27.32 ± 1.98	29.23 ± 2.29	32.38 ± 3.76	<0.001
NC, cm		37.95 ± 3.50	39.70 ± 3.31	41.01 ± 3.60	42.61 ± 3.45	<0.001
WC, cm		92.47 ± 8.02	98.33 ± 7.17	102.86 ± 7.46	109.95 ± 10.18	<0.001
Baseline SBP, mmHg		133.84 ± 16.25	133.51 ± 15.35	134.45 ± 15.47	133.80 ± 15.98	0.762
Baseline DBP, mmHg		85.00 ± 11.05	84.80 ± 11.10	85.86 ± 10.68	85.64 ± 11.26	0.276
Baseline heart rate, bpm		76.36 ± 9.14	75.86 ± 9.23	76.26 ± 8.98	75.99 ± 9.67	0.759
ALT, U/L		18.31 ± 12.75	23.59 ± 11.74	29.25 ± 16.63	43.57 ± 27.16	<0.001
AST, U/L		21.66 ± 22.53	20.91 ± 8.97	21.70 ± 10.24	25.26 ± 12.39	<0.001
Cr, µmol/L		75.54 ± 20.99	76.74 ± 20.31	77.27 ± 18.31	79.28 ± 19.85	0.010
BUN, mmol/L		5.28 ± 1.56	5.39 ± 1.53	5.31 ± 1.48	5.37 ± 1.53	0.534
TC, mmol/L		4.56 ± 1.09	4.49 ± 0.93	4.56 ± 1.20	4.59 ± 1.47	0.510
TG, mmol/L		2.16 ± 1.70	2.18 ± 1.57	2.21 ± 1.88	2.17 ± 1.56	0.963
HDL-C, mmol/L		1.11 ± 0.29	1.10 ± 0.28	1.12 ± 0.31	1.09 ± 0.29	0.204
LDL-C, mmol/L		2.64 ± 0.84	2.62 ± 0.79	2.62 ± 0.75	2.67 ± 0.81	0.616
Hcy, mmol/L		18.41 ± 11.21	17.63 ± 10.47	17.89 ± 10.70	18.45 ± 10.73	0.466
AHI, events/hour		24.45 ± 19.24	24.80 ± 19.40	24.24 ± 19.41	26.27 ± 20.73	0.262
Mean ${\mathrm{SaO}}_{2}$, %		91.67 ± 3.89	91.77 ± 3.79	91.80 ± 2.88	91.87 ± 2.85	0.771
Lowest ${\mathrm{SaO}}_{2}$, %		77.56 ± 9.25	77.41 ± 8.72	77.93 ± 8.71	77.52 ± 9.28	0.758
Smoking status, n (%)						0.010
	Never	371 (60.23%)	361 (58.51%)	341 (55.27%)	340 (55.11%)	
	Past	76 (12.34%)	66 (10.70%)	56 (9.08%)	54 (8.75%)	
	Current	169 (27.44%)	190 (30.79%)	220 (35.66%)	223 (36.14%)	
Drinking status,n (%)						0.031
	Never	401 (65.10%)	377 (61.10%)	345 (55.92%)	369 (59.81%)	
	Past	38 (6.17%)	41 (6.65%)	48 (7.78%)	55 (8.91%)	
	Current	177 (28.73%)	199 (32.25%)	224 (36.30%)	193 (31.28%)	
OSA grade, n (%)						0.608
	Mild	247 (40.10%)	243 (39.38%)	243 (39.38%)	229 (37.12%)	
	Moderate	181 (29.38%)	170 (27.55%)	192 (31.12%)	180 (29.17%)	
	Severe	188 (30.52%)	204 (33.06%)	182 (29.50%)	208 (33.71%)	
Duration of hypertension, years		6.23 ± 7.63	6.26 ± 6.81	5.85 ± 6.63	6.18 ± 6.68	0.709
History of CVD, n (%)		76 (12.34%)	66 (10.70%)	77 (12.48%)	81 (13.13%)	0.603
History of diabetes, n (%)		42 (6.82%)	85 (13.78%)	131 (21.23%)	190 (30.79%)	<0.001
History of stroke, n (%)		250 (40.58%)	216 (35.01%)	193 (31.28%)	175 (28.36%)	<0.001
Medication use, n (%)						
	ACEI/ARB	263 (42.69%)	287 (46.52%)	292 (47.33%)	302 (48.95%)	0.156
	β	128 (20.78%)	109 (17.67%)	127 (20.58%)	121 (19.61%)	0.498
	CCB	365 (59.25%)	384 (62.24%)	375 (60.78%)	366 (59.32%)	0.671
	Diuretic	47 (7.63%)	71 (11.51%)	69 (11.18%)	61 (9.89%)	0.096
	Hypoglycemic drugs	136 (22.08%)	118 (19.12%)	136 (22.04%)	133 (21.56%)	0.535
	Antiplatelet drugs	199 (32.31%)	217 (35.17%)	223 (36.14%)	216 (35.01%)	0.530
	Lipid-lowering drugs	204 (33.12%)	225 (36.47%)	218 (35.33%)	227 (36.79%)	0.526

Notes: Values are presented as mean ± SD or n (%). Abbreviations: HSI, hepatic steatosis index; SBP, systolic blood pressure; DBP, diastolic blood pressure; BMI, body mass index; NC, neck circumference; WC, waist circumference; AST, aspartate transaminase; ALT, alanine transaminase; Cr, creatinine; BUN, blood urea nitrogen; TG, triglyceride; TC, total cholesterol; LDL-C, low-density lipoprotein cholesterol; HDL-C, high-density lipoprotein cholesterol; Hcy, homocysteine; AHI, apnea–hypopnea index; mean ${\mathrm{SaO}}_{2}$, mean oxygen saturation; lowest ${\mathrm{SaO}}_{2}$, lowest oxygen saturation; CVD, cardiovascular diseases; OSA, obstructive sleep apnea; ACEI, angiotensin-converting enzyme inhibitor; ARB, angiotensin receptor blocker; CCB, calcium channel blocker; β, β-receptor blocker.

### 3.2 Increased Hepatic Steatosis Index Values Were Associated with Increased Survival Risk and Decreased Survival Time in Hypertensive Patients with Obstructive Sleep Apnea

**Fig. 2.**
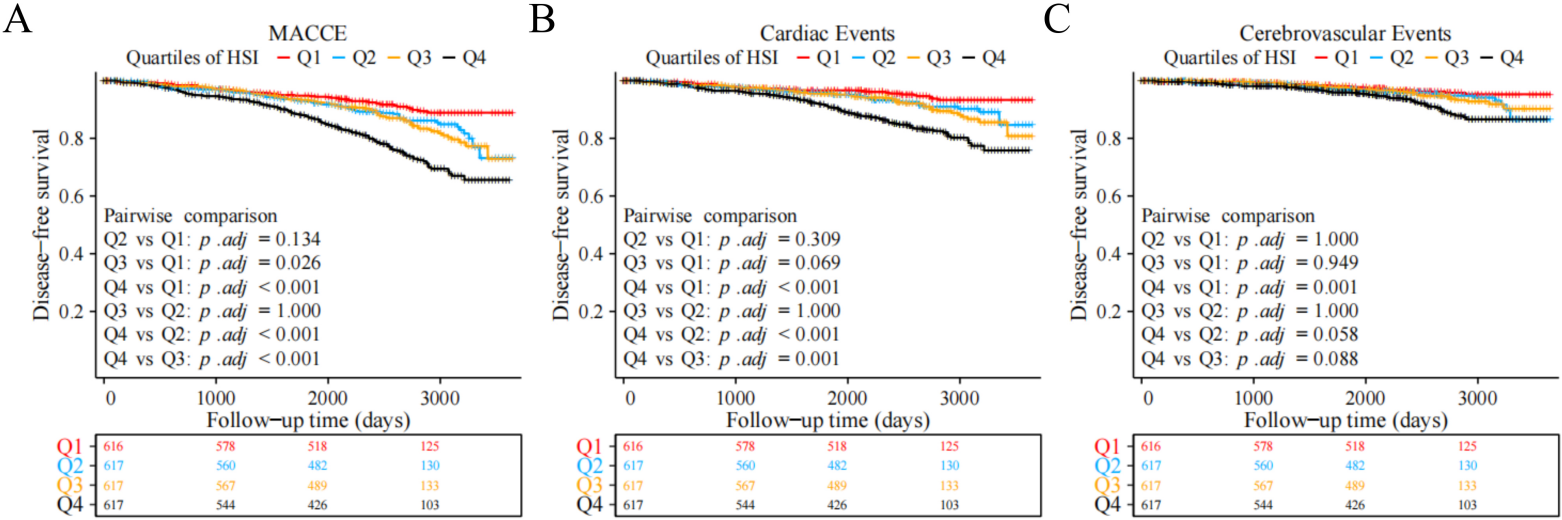
**Kaplan–Meier survival curves of different endpoints across HSI quartile groups.** (A) MACCE. (B) Cardiac events. (C) Cerebrovascular events. Abbreviations: HSI, hepatic steatosis index; MACCE, major adverse cardiovascular and cerebrovascular events.

The Kaplan–Meier curve showed that the risks of MACCE, cardiac events, and cerebrovascular events in group Q4 were significantly higher than those in the other groups (log-rank test, *p <* 0.001). Additional pairwise comparisons between groups revealed that the risk of MACCE in group Q4 was significantly higher than those in groups Q1–Q3 (adjusted *p* < 0.001), and the risk in group Q3 was also significantly higher than that in group Q1 (adjusted *p *= 0.026), although no significant differences were observed in the risks between groups Q1 and Q2 and between groups Q2 and Q3. For cardiac events, except for comparisons with group Q4, the other three groups showed no statistically significant differences (adjusted *p* > 0.05). For cerebrovascular events, the risk in group Q4 was still higher than those in the other groups, but the risk of cerebrovascular events did not differ significantly among the other groups (Fig. 2).

**Fig. 3.**
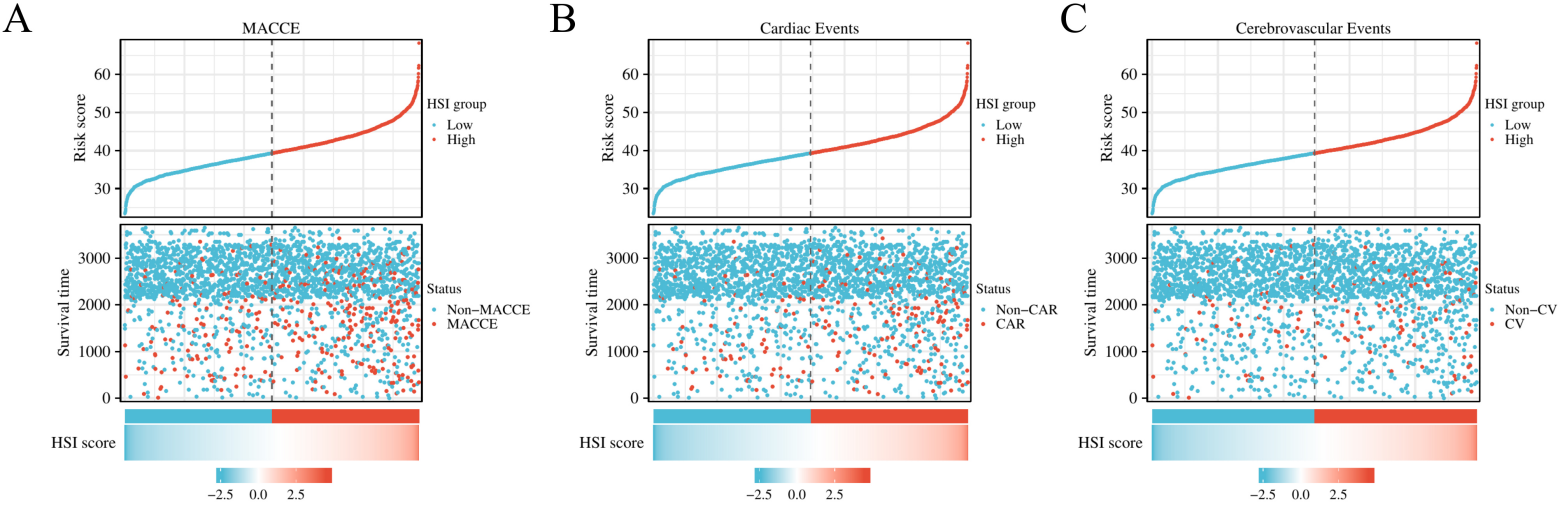
**Risk-factor plot using continuous HSI values and different endpoints.** (A) MACCE. (B) Cardiac events. (C) Cerebrovascular events. Abbreviations: HSI, hepatic steatosis index; MACCE, major adverse cardiovascular and cerebrovascular events. Non-MACCE, patients with no MACCE; CAR, patients with cardiac events; Non-CAR, patients with no cardiac events; CV, patients with cerebrovascular events; Non-CV, patients with no cerebrovascular events.

We used the continuous variable value of the HSI as the risk score, divided the population into low-risk and high-risk groups on the basis of the median, and drew risk-factor maps for MACCE, cardiac events, and cerebrovascular events. The results showed that during the median follow-up period of 81.96 months, 130 MACCE, 78 cardiac events, and 52 cerebrovascular events occurred in the low-risk group and 226 MACCE, 145 cardiac events, and 81 cerebrovascular events occurred in the high-risk group. As the HSI value increased, the frequencies of MACCE (5.3% vs. 9.2%, *p <* 0.001), cardiac events (3.2% vs. 5.9%, *p <* 0.001), and cerebrovascular events (2.1% vs. 3.3%, *p *= 0.013) also increased (**Supplementary Table 1**, Fig. 3), while the survival duration decreased (81.96 months vs. 81.00 months, *p *= 0.012. **Supplementary Table 1**). Univariate Cox analysis showed that the risks of MACCE (HR [hazard ratio], 1.829; 95% CI [confidence interva]: 1.474–2.270; *p <* 0.001), cardiac events (HR, 1.954; 95% CI: 1.484–2.573; *p <* 0.001), and cerebrovascular events (HR, 1.642; 95% CI: 1.159–2.326; *p *= 0.005) were higher in the HSI high-risk group than in the low-risk group (**Supplementary Table 2**).

### 3.3 Hepatic Steatosis Index Shows a Linear Relationship with the Risk of Endpoint Events

Before developing the Cox model, we assessed the collinearity of the covariates using the variance inflation factor (VIF). A VIF >5 was considered to indicate a covariate with substantial collinearity; such covariates were eliminated during the second screening round and eventually not included in the Cox model (**Supplementary Table 3**). Using the HSI quartile as a categorical variable to establish a Cox proportional-hazards model, we determined whether the risk of MACCE in the Q2–Q4 (HSI >35.5) groups was significantly (*p <* 0.05) higher than that in the Q1 group in the original or adjusted models (Table 2). With multi-factor adjustment, the HR value for MACCE increased from 1.50 (95% CI: 1.06–2.13; *p *= 0.023) to 1.63 (95% CI: 1.09–2.44; *p *= 0.017) in the Q2 group; from 1.64 (95% CI: 1.17–2.31, *p *= 0.005) to 1.71 (95% CI: 1.14–2.55, *p *= 0.009) in the Q3 group; and from 2.99 (95% CI: 2.18–4.11; *p <* 0.001) to 2.95 (95% CI: 1.99–4.39; *p <* 0.001) in the Q4 group. Similar results were obtained for cardiac and cerebrovascular events. In the multivariate adjustment model, in comparison with the Q1 group, the HRs of cardiac events in groups Q2, Q3, and Q4 were 1.64 (95% CI: 0.98–2.74; *p *= 0.061), 1.75 (95% CI: 1.05–2.92; *p *= 0.033), and 2.80 (95% CI: 1.68–4.66; *p <* 0.001), respectively (**Supplementary Table 3**); the corresponding HRs for cerebrovascular events were 1.63 (95% CI: 0.85–3.12; *p *= 0.140), 1.63 (95% CI: 0.85–3.12; *p *= 0.137), and 3.21 (95% CI: 1.71–6.03; *p <* 0.001) (**Supplementary Table 4**), respectively. A Cox model was established using the HSI as a continuous variable. In the original model, for every 1-SD increase in the HSI, the risks of MACCE, cardiac events, and cerebrovascular events increased by 49%, 54%, and 40%, respectively. After adjusting for multiple factors, the risks rose to 43% (Table 2), 38% (**Supplementary Table 4**), and 51% (**Supplementary Table 5**), respectively.

**Table 2.** **Relationship between HSI and MACCE in different models**.

Variable		Non-adjusted		Adjusted model I		Adjusted model II	
		HR (95% CI)	*p*-value	HR (95% CI)	*p*-value	HR (95% CI)	*p*-value
HSI (Per 1 SD increase)		1.49 (1.35, 1.64)	<0.001	1.57 (1.42, 1.74)	<0.001	1.43 (1.26, 1.62)	<0.001
Quartiles of HSI							
	Q1	1.0		1.0		1.0	
	Q2	1.50 (1.06, 2.13)	0.023	1.60 (1.12, 2.28)	<0.001	1.63 (1.09, 2.44)	0.017
	Q3	1.64 (1.17, 2.31)	0.005	1.82 (1.28, 2.58)	<0.001	1.71 (1.14, 2.55)	0.009
	Q4	2.99 (2.18, 4.11)	<0.001	3.44 (2.48, 4.78)	<0.001	2.95 (1.99, 4.39)	<0.001

Abbreviations: SBP, systolic blood pressure; DBP, diastolic blood pressure; Hcy, homocysteine; OSA, obstructive sleep apnea; HSI, hepatic steatosis index; MACCE, major adverse cardiovascular and cerebrovascular events; HR, hazard ratio; 95% CI, 95% confidence interval. The non-adjusted model did not include adjustments for any factor. Adjusted model I was adjusted for age and sex. Adjusted model II was adjusted for age, sex, history of diabetes, smoking status, alcohol consumption status, baseline SBP, baseline DBP, baseline heart rate, Hcy level, and OSA grade.

**Fig. 4.**
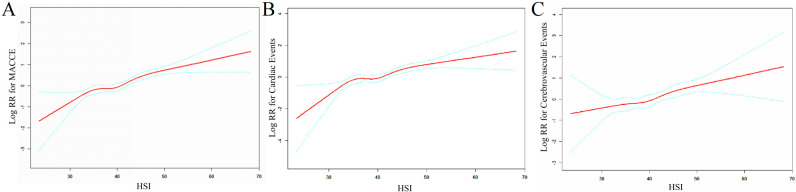
**Dose–response relationship between the HSI and the risk of different endpoint events. **(A) MACCE; (B) cardiac events; (C) cerebrovascular events. Abbreviations: RR, relative risk; HSI, hepatic steatosis index; MACCE, major adverse cardiovascular and cerebrovascular events.

To clarify the potential relationship between continuous increments in HSI values and endpoint events, we further established a generalized additive model with adjustments for age, sex, baseline systolic blood pressure, diastolic blood pressure, heart rate, BMI, Hcy level, smoking and alcohol consumption status, OSA classification, and history of diabetes, and the results showed that the HSI was linearly associated with the occurrence of endpoint events, especially for cerebrovascular events (Fig. 4).

### 3.4 Influence of Hepatic Steatosis Index Increments on the Predictive Performance for Endpoint Events in Hypertensive Patients with Obstructive Sleep Apnea

Table 3 shows that in comparison with the traditional model (adjusted for age, sex, smoking status, alcohol consumption status, history of diabetes, SBP, DBP, heart rate, Hcy level, OSA grade at the baseline), the discriminant and classification performances of the model with the HSI were higher for endpoint events. After adding the HSI, for MACCE events, the C-statistic increased from 0.609 (95% CI: 0.578–0.640) to 0.649 (95% CI: 0.618–0.680; *p <* 0.001), IDI was 0.030 (*p <* 0.001), and the continuous NRI was 0.153 (*p <* 0.001). For cardiac events, the C-statistic increased from 0.643 to 0.672 (*p* < 0.001), IDI was 0.024 (*p <* 0.001), and continuous NRI was 0.136 (*p <* 0.001). For cerebrovascular events, the C-statistic increased from 0.608 to 0.641 (*p <* 0.001), IDI was 0.015 (*p <* 0.001), and continuous NRI was 0.170 (*p* = 0.002).

**Table 3.** **C-statistic and reclassification discriminant analysis of the HSI for different endpoint events**.

		C-Statistic (95% CI)	*p*-value	IDI	*p*-value	Continuous-NRI	*p*-value
MACCE							
	Conventional model	0.609 (0.578, 0.640)	—	Ref	—	Ref	—
	Conventional model + HSI	0.649 (0.618, 0.680)	<0.001	0.030 (0.016, 0.048)	<0.001	0.153 (0.087, 0.228)	<0.001
Cardiac events							
	Conventional model	0.643 (0.606, 0.680)	—	Ref	—	Ref	—
	Conventional model + HSI	0.672 (0.632, 0.711)	<0.001	0.024 (0.011, 0.044)	<0.001	0.136 (0.062, 0.237)	<0.001
Cerebrovascular event							
	Conventional model	0.608 (0.555, 0.661)	—	Ref	—	Ref	—
	Conventional model + HSI	0.641 (0.590, 0.692)	<0.001	0.015 (0.004, 0.038)	<0.001	0.170 (0.087, 0.282)	0.002

Notes: The conventional model was adjusted for age, sex, smoking status, alcohol consumption status, history of diabetes, systolic blood pressure, diastolic blood pressure, heart rate, homocysteine level, and obstructive sleep apnea grade at baseline. Abbreviations: IDI, integrated discrimination improvement; NRI, net reclassification index; HSI, hepatic steatosis index; MACCE, major adverse cardiovascular and cerebrovascular events.

## 4. Discussion

China has a large population of hypertensive patients with OSA, and this population often shows multiple risk factors that promote each other and further increase the risk of CVD and serious cardiovascular adverse events [28, 29]. However, the current clinical guidelines do not include assessments of serious cardiovascular adverse events in hypertensive patients with OSA. Therefore, this study used a retrospective cohort to characterize the relationship between the HSI and MACCE in a hypertensive population with OSA. Our results showed that an increase in the HSI value was independently associated with an increased risk of MACCE. The risk of MACCE in the highest HSI quartile was 2.95 times that in the lowest quartile. We observed similar results and trends when the study endpoints were subdivided into cardiac and cerebrovascular events and observed a linear relationship between the HSI and the risk of MACCE. More importantly, in comparison with the traditional model composed of risk factors, addition of the HSI was shown to increase the predictive power for MACCE.

Our results showed that patients with higher HSI values had a greater likelihood of MACCE. This outcome remained unchanged irrespective of whether the HSI was considered a categorical or continuous variable. In a cross-sectional study of healthy individuals, Kweon *et al*. [14] reported that participants with HSI >36 had a 2.43-fold higher risk of being in a cardiovascular high-risk group than those with HSI <30. This is consistent with the results of our study, in which patients with HSI >35.5 indicated a higher risk of MACCE. Hepatic steatosis is thought to be the component most closely associated with these results. Fatty liver has been previously shown to be an independent risk factor for CVD, and nonalcoholic fatty liver is the most common form of fatty liver [30, 31, 32, 33]. According to a meta-analysis conducted by Targher *et al*. [34], nonalcoholic fatty liver disease (NAFLD) is related to a 64% increased risk of cardiovascular disease. To further investigate the relationship between the HSI and cardiovascular and cerebrovascular disorders, the researchers developed a generalized additive model; their results revealed a linear relationship between the HSI and the risk of MACCE events. The HSI value has also been demonstrated to be linearly connected to the occurrence of cardiovascular and cerebrovascular events in the general population, and the risk of cardiovascular and cerebrovascular events increases by 4% for each 1-SD increase in the HIS [35]. Nevertheless, these findings were derived from investigations of broader populations. Our findings imply that the HSI value is associated with an increased risk of cardiovascular and cerebrovascular illnesses in patients with hypertension and OSA, which were attributable to the following reasons: First, the HSI formula includes numerous classic cardiovascular disease risk variables that have a synergistic effect on the HSI score; therefore, the association between the HSI and CVD is stronger than those of individual risk factors. Second, the higher the HSI value, the more severe the degree of hepatic steatosis. Numerous studies have indicated that OSA shows a high comorbidity rate with hypertension and that this population has a greater risk of developing lipid metabolism disorders and NAFLD than patients with simple hypertension or OSA [18, 19, 20, 21, 23]. In addition, patients with hypertension alone or those with OSA alone have a high risk of developing NAFLD [18, 33, 36], which are all independent risk factors for CVD [2, 30, 37, 38]. Third, hypertension, OSA, and NAFLD are independently associated risk factors for each other. In addition to the pathophysiological mechanisms described in the introduction by which hypertension and OSA contribute to each other, interactions between OSA and NAFLD have also been demonstrated. Bahr *et al*. [39] found a high prevalence of NAFLD in patients with moderate-to-severe OSA in a Caucasian population, while similar findings have been reported in Asian populations [40, 41]. Chung *et al*. [42] also observed a high risk of OSA in patients with NAFLD, and similar findings showed significance in special populations such as women and children, wherein NAFLD was a risk factor independent of OSA [43, 44]. Fourth, inflammatory responses and oxidative stress and structural changes in blood vessels are pathophysiological mechanisms shared by hypertension, OSA, and fatty liver, all of which can cause the vascular endothelial cell deterioration that, in combination with inflammatory factors and lipid molecules, ultimately results in adverse cardiovascular events [38, 45, 46].

The HSI was originally a noninvasive index used to assess nonalcoholic fatty liver disease, and higher HSI values indicated more pronounced hepatic steatosis [12]. In addition to being directly associated with a nonalcoholic fatty liver, the HSI is also intimately associated with the development and occurrence of other disorders. Cai *et al*. [13] discovered that a 1-SD increase in the HSI in a healthy group was related to a 62% greater chance of developing diabetes. In addition, Han *et al*. [47] found that dynamic monitoring of the HSI can more accurately predict the onset of diabetes. The HSI is also independently linked to decreased bone mineral density, dementia, asthma, and sarcopenia [48, 49, 50, 51]. These studies have shown that the HSI can not only be used as a predictor of adverse diseases, but its ability to predict adverse events is not disease-specific. Therefore, we utilized the HSI reliably to assess CVD risk. In addition, we assessed the discriminative performance of the model after incorporating the HSI by evaluating the C-statistic, NRI, and IDI, and using other approaches, and the assessments yielded ideal outcomes.

This study had the following strengths. First, it was a retrospective cohort study with a large sample size of a special population with hypertension and OSA, and the results were relatively stable and reliable after statistical adjustments for confounding variables. Second, after adjusting for multiple confounding variables, HSI was independently associated with MACCE in the hypertensive OSA population. Third, using the C-statistic, IDI, and NRI, our findings validated the superior discriminatory ability of the model including the HSI in comparison with the conventional model, lending credence to the notion that the HSI is a better predictor for patients with hypertension and OSA. However, this study also had the following limitations: First, this was a retrospective single-center cohort study that was subject to the limitations of nonrandomized trials; therefore, the results should be interpreted with caution. However, every effort was made to ensure the reliability of the findings by adjusting for confounding factors using statistical methods. Second, we only investigated the association between the HSI and MACCE at baseline; however, factors such as medication, lifestyle, weight changes, and the presence or absence of CPAP treatment during patient follow-up could contribute to reverse causality. Consequently, our next line of inquiry will focus on time-dependent HSI variations and the associated influencing factors. Third, the study population consisted of patients with hypertension and OSA from western China, and the prevalence of hypertension, OSA, and CVD can be affected by local diet, lifestyle, and ethnicity, which can strengthen or weaken the association between the HSI and MACCE. Therefore, the findings of this study are not applicable to other populations, but they indicate the need for long-term clinical evaluation of specific patient populations, such as those with hypertension with OSA. Fourth, the paucity of liver-related imaging data in this study precluded further imaging-based evaluation of the severity of hepatic steatosis in patients. Fifth, genetic background can also influence hypertension, OSA, metabolic diseases, and CVD; however, our study did not evaluate genetic factors; therefore, the use of methods such as Mendelian randomization may provide new genetic-level insights into the observed results.

## 5. Conclusions

Among patients showing a combination of hypertension and OSA, those with high HSI were at an increased risk of CVD, and HSI showed a linear relationship with the risk of MACCE. Therefore, patients showing hypertension combined with OSA should undergo HSI evaluations as early as possible, and early intervention on the basis of the HSI levels should be provided to prevent adverse cardiovascular events.

## Data Availability

The data of this study are available from the corresponding author upon reasonable request.

## Supplementary Material

Supplementary material associated with this article can be found, in the online version, at https://doi.org/10.31083/j.rcm2410280.
