# Supplementary material for: Linear Relationship between Hepatic Steatosis Index and Major Adverse Cardiovascular Events in Hypertensive Patients with Obstructive Sleep Apnea: A Real-World Cohort Study from China
Source: Rev Cardiovasc Med. 2023 Oct 7;24(10):280. doi: 10.31083/j.rcm2410280 (PMC11273115; doi:10.31083/j.rcm2410280)
Supplement: Supplementary file 1 [file 2153-8174-24-10-280-s1.docx]

Supplementary Material

# Supplementary Tables

**Table S1**. Comparison of the frequency of end-point events and differences in survival time between the two groups grouped by the median of HSI.

| Characteristic | Low risk group | High risk group | *P*-value |
| --- | --- | --- | --- |
|  | n=1233 | n=1234 |  |
| MACCE, n (%) | 130 (5.3%) | 226 (9.2%) | < 0.001 |
| Cardiac events, n (%) | 78 (3.2%) | 145 (5.9%) | < 0.001 |
| Cerebrovascular events, n (%) | 52 (2.1%) | 81 (3.3%) | 0.013 |
| Survival time(months), median (IQR) | 81.96 (72, 96) | 81 (63.96, 95.04) | 0.012 |

Annotation: MACCE, Major adverse cardiovascular and cerebrovascular events; HSI, hepatic steatosis index.

**Table S2**. Univariate cox analysis of HSI grouped by median in different endpoint events

| Variable | Hazard ratio (95% CI) | *P*-value |
| --- | --- | --- |
| **MACCE** |  |  |
| HSI low risk group | Ref | Ref |
| HSI high risk group | 1.829 (1.474-2.270) | **<0.001** |
| **Cardiac events** |  |  |
| HSI low risk group | Ref | Ref |
| HSI high risk group | 1.954 (1.484-2.573) | **<0.001** |
| **Cerebrovascular Events** |  |  |
| HSI low risk group | Ref | Ref |
| HSI high risk group | 1.642 (1.159-2.326) | **0.005** |

Annotation: MACCE, Major adverse cardiovascular and cerebrovascular events; HSI, hepatic steatosis index; HR, hazard ratio; 95%CI, 95% confidence interval.

**Table S3**. Collinearity diagnosis of independent variables using variance inflation factor.

| Variable | VIF 1 | VIF 2 |
| --- | --- | --- |
| HSI | 7.618 | 3.728 |
| Age | 1.897 | 1.896 |
| Sex | 2.600 | 2.579 |
| BMI | 6.611 | NA |
| NC | 2.666 | 2.610 |
| WC | 3.312 | 2.672 |
| Baseline SBP | 2.112 | 2.111 |
| Baseline DBP | 2.280 | 2.279 |
| Baseline heart rate | 1.055 | 1.055 |
| ALT | 4.824 | 3.531 |
| AST | 2.969 | 2.484 |
| Cr | 1.567 | 1.566 |
| BUN | 1.265 | 1.264 |
| TC | 1.833 | 1.833 |
| TG | 1.311 | 1.311 |
| HDL-C | 1.212 | 1.211 |
| LDL-C | 1.629 | 1.628 |
| Hcy | 1.050 | 1.046 |
| AHI | 3.954 | 3.944 |
| Mean SaO2 | 1.290 | 1.290 |
| Lowest SaO2 | 1.745 | 1.743 |
| Smoking status | 1.639 | 1.634 |
| Drinking status | 1.521 | 1.521 |
| OSA grade | 3.526 | 3.522 |
| Duration of hypertension | 1.343 | 1.337 |
| History of CVD | 1.133 | 1.337 |
| History of diabetes | 1.326 | 1.227 |
| History of stroke | 1.169 | 1.166 |

Abbreviations: VIF, variance inflation factor; HSI, hepatic steatosis index; SBP, systolic blood pressure; DBP, diastolic blood pressure; BMI, body mass index; NC, neck circumference; WC, waist circumference; AST, aspartate transaminase; ALT, alanine transaminase; Cr, creatinine; BUN, blood urea nitrogen; TG, triglyceride; TC, total cholesterol; LDL-C, low-density lipoprotein cholesterol; HDL-C, high-density lipoprotein cholesterol; Hcy, homocysteine; AHI, apnea-hypopnea index; mean SaO_2_, mean oxygen saturation; lowest SaO_2_, lowest oxygen saturation; CVD, cardiovascular diseases; OSA, obstructive sleep apne

**Table S4**. Relationship between HSI and cardiac event in different models

| Variable | Non-adjusted | | Adjust model I | | Adjust model II | |
| --- | --- | --- | --- | --- | --- | --- |
|  | HR (95% CI) | *P*-value | HR (95% CI) | *P*-value | HR (95% CI) | *P*-value |
| HSI (Per 1 SD increase) | 1.54 (1.36, 1.74) | <0.001 | 1.64 (1.44, 1.86) | <0.001 | 1.38 (1.18, 1.62) | <0.001 |
| Quartiles of HSI |  |  |  |  |  |  |
| Q1 | 1.0 |  | 1.0 |  | 1.0 |  |
| Q2 | 1.56 (0.99, 2.46) | 0.053 | 1.68 (1.06, 2.65) | 0.027 | 1.64 (0.98, 2.74) | 0.061 |
| Q3 | 1.76 (1.13, 2.74) | 0.012 | 1.98 (1.26, 3.10) | 0.003 | 1.75 (1.05, 2.92) | 0.033 |
| Q4 | 3.31 (2.20, 4.98) | <0.001 | 3.87 (2.53, 5.91) | <0.001 | 2.80 (1.68, 4.66) | <0.001 |
| *P* for trend | - | <0.001 | - | <0.001 | - | <0.001 |

Annotation: SBP, systolic blood pressure; DBP, diastolic blood pressure; Hcy, homocysteine; OSA, obstructive sleep apnea; HSI, hepatic steatosis index; HR, hazard ratio; 95%CI, 95% confidence interval.

Non-adjusted model adjust for: None

Adjust model I adjust for: Age; Sex

Adjust model II adjust for: Age; Sex; History of diabetes; Smoking status; Drinking status; Baseline SBP; Baseline DBP; Baseline heart rate; Hcy; OSA grade.

**Table S5**. Relationship between HSI and cerebrovascular event in different models

| Variable | Non-adjusted | | Adjust model I | | Adjust model II | |
| --- | --- | --- | --- | --- | --- | --- |
|  | HR (95% CI) | *P*-value | HR (95% CI) | *P*-value | HR (95% CI) | *P*-value |
| HSI (Per 1 SD increase) | 1.40 (1.19, 1.65) | <0.001 | 1.47 (1.24, 1.73) | <0.001 | 1.51 (1.23, 1.86) | <0.001 |
| Quartiles of HSI |  |  |  |  |  |  |
| Q1 | 1.0 |  | 1.0 |  | 1.0 |  |
| Q2 | 1.41 (0.81, 2.44) | 0.223 | 1.50 (0.86, 2.61) | 0.152 | 1.63 (0.85, 3.12) | 0.140 |
| Q3 | 1.47 (0.85, 2.53) | 0.165 | 1.60 (0.92, 2.79) | 0.094 | 1.63 (0.85, 3.12) | 0.137 |
| Q4 | 2.54 (1.53, 4.20) | <0.001 | 2.87 (1.70, 4.83) | <0.001 | 3.21 (1.71, 6.03) | <0.001 |
| *P* for trend | - | <0.001 | - | <0.001 | - | <0.001 |

Annotation: SBP, systolic blood pressure; DBP, diastolic blood pressure; Hcy, homocysteine; OSA, obstructive sleep apnea; HSI, hepatic steatosis index; HR, hazard ratio; 95%CI, 95% confidence interval.

Non-adjusted model adjust for: None

Adjust model I adjust for: Age; Sex

Adjust model II adjust for: Age; Sex; History of diabetes; Smoking status; Drinking status; Baseline SBP; Baseline DBP; Baseline heart rate; Hcy; OSA grade.
